# The nitric oxide synthase 3 G894T polymorphism associated with Alzheimer’s disease risk: a meta-analysis

**DOI:** 10.1038/srep13598

**Published:** 2015-09-04

**Authors:** Shengyuan Liu, Fangfang Zeng, Changyi Wang, Zhongwei Chen, Bin Zhao, Keshen Li

**Affiliations:** 1 Guangdong Key Laboratory of Age-Related Cardiac and Cerebral Diseases, Affiliated Hospital of Guangdong Medical College, Zhanjiang 524001, China; 2 Department of Chronic Disease, Shenzhen Nanshan Center for Chronic Disease Control, Shenzhen 518054, China; 3 Epidemiology research unit, The First Affiliated Hospital of Sun Yat-sen University, Guangzhou, Guangdong, 510080, China; 4 Institute of Neurology, Guangdong Medical College, Zhanjiang 524001, China

## Abstract

The association between the G894T polymorphism (Glu298Asp) of nitric oxide synthase 3 (*NOS3*) and risk of Alzheimer’s disease (AD) was explored by performing a meta-analysis of case-control studies. Bibliographical searches were conducted in the MEDLINE, EMBASE, and China National Knowledge Infrastructure (CNKI) databases without any language limitations. Two investigators independently assessed abstracts for relevant studies, and reviewed all eligible studies. We adopted regrouping in accordance with the most probably appropriate genetic model. Odds ratios (ORs) with 95% confidence intervals (CIs) were used to assess the strength of this association. We performed a meta-analysis including 21 published articles with 23 case-control studies (5,670 cases and 5,046 controls). In the analyses, we found significant association between G894T polymorphism and AD risk under a complete overdominant model (GG + TT vs. GT) (OR = 1.18; 95%CI, 1.04–1.35; P = 0.010). When stratified by time of AD onset, we found the association between this polymorphism and AD susceptibility to be more substantial among late onset patients than among early onset patients (OR for late vs. early onset: 1.33 vs. 1.02, P interaction = 0.049). The meta-analysis showed that the polymorphism G894T of *NOS3* was associated with risk of AD.

Alzheimer’s disease (AD), also called senile dementia of the Alzheimer type or primary degenerative dementia of the Alzheimer’s type, is a degenerative disease of the central nervous system characterized by a gradual decline in memory and cognition, which has been correlated with synaptic dysfunction and loss, and eventually to neuronal death^1^. According to data from the World Alzheimer Report, the number of people with AD is forecast to nearly double every 20 years from 36 million in 2010 to 115 million in 2050, and the costs associated with AD reached the total of US$604 billion, about 1% of global GDP^2^. Therefore, it is particularly urgent to gain an insight into the pathogenesis factors of the AD in order to discover different possibilities of preventive and effective treatment.

Although it was not until 1901 that the first case with AD was identified in a fifty-year-old woman by German psychiatrist Alois Alzheimer^3^, its pathological cause, like many other mental diseases, is unclear so far. Recently, it is reported that oxidative stress caused by nitric oxide (NO) may play a pathogenetic role in AD. The deposits of β-Amyloid (Aβ) in AD brains can lead to the production of superoxide radicals. These radicals combined with NO in endothelial cells to form peroxynitrite, which in turn induced cellular injury and caused lipid peroxidation to further accelerate neurodegenerative changes leading to AD incurrence^4^. NO in endothelial cells mainly was synthesized from L-arginine by NOS3. Therefore, NOS3 may be a pivotal protein involved in a complex cascade of events in the oxidative stress.

*NOS3*, gene encoding for nitric oxide synthase 3, is located on chromosome 7q35^5^ and has been widely studied in several AD populations. Dahiyat *et al.*^6^ first found a significant effect of the G allele and the GG genotype of *NOS3* G894T polymorphism on AD development. Subsequently, this association has been studied in many studies^7^,^8^, but with inconsistent findings. Therefore, we conducted a meta-analysis of the existing epidemiologic studies by using a comprehensive search strategy to determine whether there was an association between G894T polymorphism of *NOS3* and risk of AD.

## Material and Methods

### Study selection

Methodology advocated by the MOOSE guideline was followed to perform this meta-analysis^9^. We conducted a systematically electronic search using the following terms “(Alzheimer* or AD) and (NOS* or nitric oxide synthase*) and (polymorphism* or genotype* or variant*)” in the PubMed database (from 1966 to May, 2015). We subsequently repeated this search in the EMBASE, CNKI (http://www.cnki.net) and GOOGLE Scholar (http://scholar.google.com/). Reference lists of relevant articles were reviewed manually to search for more studies.

For inclusion, studies included in the meta-analysis had to meet the following criteria: (1) studies were designed as the case–control type; (2) reported outcomes included AD; and (3) polymorphism included G894T (Glu298Asp) polymorphism. Studies were excluded if: (1) no detailed genotype frequency; and (2) insufficient information for data extraction. When multiple publications from the same patient population resource or overlapping data sets were available, only the most recent or largest sample size study was included in the meta-analysis.

### Data extraction

The citations (titles and abstracts) search and data extraction were carried out independently by two reviewers, and disagreements were resolved by consensus. The following information was collected in a predefined data collection form: the first author’s name, year of publication, country of origin, ethnicity, AD diagnosis method, source of controls, proportion of men in cases and controls, total number of cases and controls, mean (range) age of cases and controls, time of AD onset, and number of cases and controls with different genotypes. The quality of each study selected for inclusion in the meta-analysis was assessed by quality assessment criteria, which was derived from previously published meta-analysis of molecular association studies^10^. Total scores range from 0 to 15, with 15 being the maximum. Quality was assigned as optimal with 10–15 points and suboptimal with 0–9 points^11^.

Quality scores of studies ranged from 0 (lowest) to 15 (highest). Studies with scores ≤9 were categorized into low quality, while those with scores >9 were considered as high quality^11^.

### Genotype based mRNA expression analysis

We used the data of NOS3 G894T genotypes from the International HapMap Project (http://hapmap.ncbi.nlm.nih.gov/) for the 209 subjects, and their corresponding mRNA expression levels data were available from SNPexp (http://app3.titan.uio.no/biotools/help.php?app=snpexp) as described previously^12^,^13^.

### Statistical analysis

We used crude Odds ratios (ORs) with their 95% confidence intervals (CIs) to assess the strength of association between G894T polymorphism and AD risks. OR1, OR2, and OR3 were calculated for the genotypes GG vs. TT, GT vs. TT, and GG vs. GT for G894T polymorphism, respectively. The most appropriate genetic model was determined by the above pairwise differences: If OR1 = OR3 ≠ 1 and OR2 = 1, then a recessive model (GG vs. GT + TT) is suggested; If OR1 = OR2 ≠ 1 and OR3 = 1, then a dominant model (GG + GT vs. TT) is suggested; If OR2 = 1/OR3 ≠ 1 and OR1 = 1, then a complete overdominant model (GG + TT vs. GT) is suggested; If OR1 > OR2 > 1 and OR1 > OR3 > 1 (or OR1 < OR2 < 1 and OR1 < OR3 < 1), then a codominant model (GG vs. GT vs. TT) is suggested^14^. We also performed subgroup analysis according to time of AD onset, *APOE ε4* polymorphism, ethnicity, HWE in controls, control population source, year of publication, and quality score, respectively.

The departure of frequencies in controls from expectation under Hardy–Weinberg equilibrium (HWE) was assessed by chi-square test. We calculated the false-positive report probability (FPRP) to evaluate the significant findings as described previously^15^. 0.2 was set as an FPRP threshold and assigned a prior probability of 0.1 to detect an odds ratio (OR) of 0.67/1.50 (protective/risk effects) for an association with genotypes under investigation. An FPRP value <0.2 was considered as a noteworthy finding. Sensitivity analysis for the overall effect was performed by influence-analyses to evaluate whether a single or more studies might markedly affect the results^16^. Statistical heterogeneity between studies was determined by the Q-test and P < 0.05 was considered statistically significant. With lack of heterogeneity among studies, the pooled OR estimate was merged by the fixed effects model^17^. Otherwise, the random effects model was applied^18^. Publication bias was investigated with Egger’s and Begg’s regression asymmetry test and funnel plot^19^,^20^. We used independent t-test to test the differences of mRNA expression between genotypes. All statistical analyses were completed using Stata Version 11.0 (College Station, TX, USA).

## Results

### Studies characteristics

**Figure 1:**
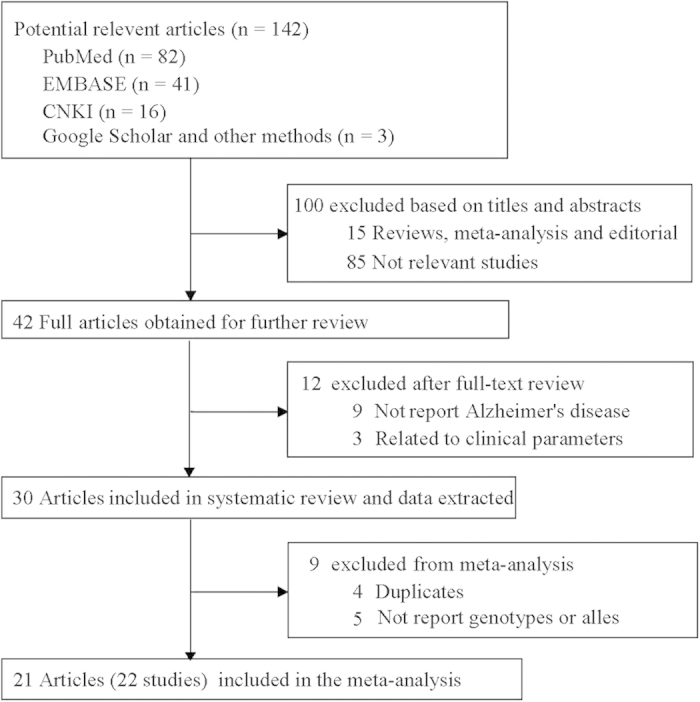
Flow chart of selection studies in our meta-analysis.

A total of 142 articles were achieved by literature search from the Pubmed, Embase, CNKI and other searching methods, using different combinations of key words (Fig. 1). 23 case-control studies from 21 publications^2^,^6^,^7^,^8^,^21^,^22^,^23^,^24^,^25^,^26^,^27^,^28^,^29^,^30^,^31^,^32^,^33^,^34^,^35^,^36^,^37^ including 5,670 cases and 5,046 controls were used to evaluate the association of G894T polymorphism with AD risk (Table 1). Sample sizes ranged from 102 to 1,369 (median 365). 17 studies included populations of Caucasian, and 6 of Asian. The mean ages at study for cases and controls were 71.4 years and 73.7 years, and the mean ages at onset of AD cases were 75.4 years. Fewer men were observed for all individual studies (percentages of men range from 25.6% to 100%, median was 38.4%). The criteria for AD diagnosis for 17 studies was the National Institute of Neurological Disorders and Stoke–Alzheimer Diseases and Related Disorders Association criteria (NINCDS/ADRDA criteria), 1 was used the fourth Diagnostic and Statistical Manual of Mental Disorders criteria (DSM-III-R criteria), and 5 for both. 9 studies only included late-onset AD cases, 2 for early-onset, and 12 for both. For most studies (n = 19), controls came from population-based settings, and 4 studies from hospital-based. The control group in 5 studies was not in HWE (Table 2).

### Quantitative synthesis

**Figure 2:**
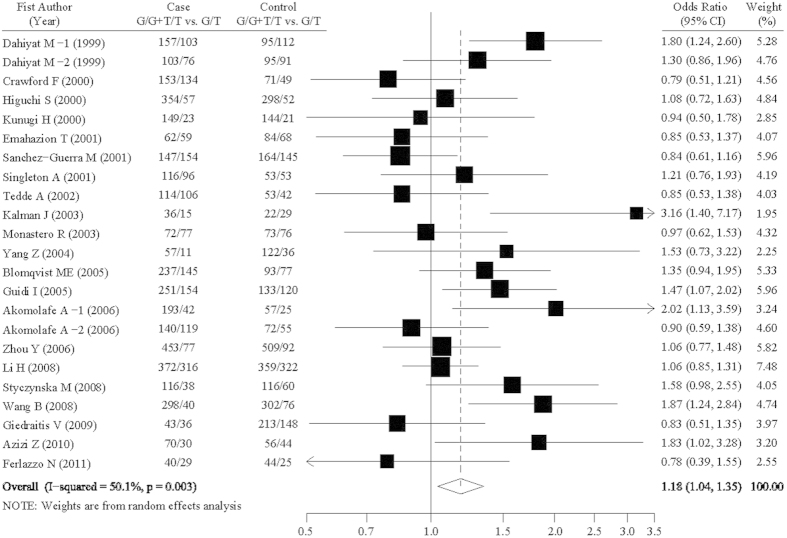
Forest plot of association between *NOS3* G894T polymorphism (G/G+T/T vs. G/T) and AD risk.

The OR1, OR2, and OR3 were 0.99 (95% CI = 0.83–1.19; P = 0.901), 0.91 (95% CI = 0.76–1.08; P = 0.289), and 1.19 (95% CI = 1.04–1.36; P = 0.011), respectively, suggesting a complete overdominant effect of the putative susceptibility allele T. Thus, in accordance with a complete overdominant model, the original grouping was collapsed, and GG and TT were merged in one group compared with the GT genotype group. The pooled odds ratios and 95% confidence intervals for the associations between G894T polymorphism and AD susceptibility was 1.18 (1.04–1.35; P = 0.010) under a complete overdominant model (GG + TT vs. GT) (Table 3, Fig. 2). For a prior probability of 0.25, the FPRP values was 0.046 with statistical power of 0.597.

When stratified by time of AD onset, we found the association between this polymorphism and AD susceptibility to be more substantial among participants with late onset of AD than among those with early onset (OR for late onset vs. early onset: 1.33 vs. 1.02, P interaction = 0.049) (Table 3). No significant interaction were observed for ethnic descent, APOE ε4 +/−, HWE in controls, control population source, time of publication, and quality score (all P interactions > 0.05).

### Sensitivity analysis and bias diagnosis

**Figure 3:**
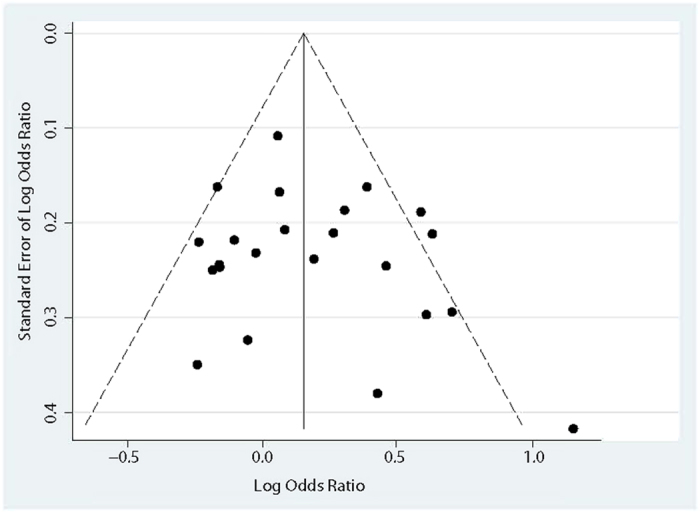
Funnel plot of association between *NOS3* G894T polymorphism (G/G+T/T vs. G/T) and the risk of AD risk.

We did sensitivity analysis to explore whether modification of included studies in the meta-analysis could influence the overall effects, and these results were unchanged in terms of magnitude and significance. The OR (95% CI) ranged from 1.16 (1.02, 1.31) to 1.21 (1.06, 1.38), which implied no other single study affected the summary risks qualitatively. The Egger’s, Begg’s test, and funnel plot were performed to access the publication bias. Ultimately, Both Egger’s and Begg’s test revealed that no significant biases existed (*P* value: 0.335 and 0.597). And the funnel plot also indicated no evidence of publication bias (Fig. 3).

### The mRNA expression by NOS3 G894T genotypes

The difference of mRNA expressions levels between genotypes were explored for three ethnic groups (i.e., CEU, YRI and Asian) and the total sample (Table 4). Significant higher mRNA expression was observed for individuals with GG genotype than those with GT genotype among YRIs (P = 0.007). No significant alteration in the mRNA expression levels was found for Caucasian, Asians, and total sample.

## Discussion

Our meta-analysis included a total of 5,670 AD case and 5,046 control subjects from 23 published case control studies for the G894T polymorphism. The results from the meta-analysis showed that there was a significant association between the polymorphism of *NOS3* and risk of AD (overdominant model: *P *< 0.001, OR = 1.18, 95%CI = 1.08–1.29), which contrasts with the results from previous meta-analyses^38^,^39^ that observed a null association between the G894T polymorphism and AD risk. Several reasons may explain the different results: First, our meta-analysis included more case-control studies than the previous meta-analysis, thus our study was more powerful and the conclusion may be more reliable. Second, we selected an optimal genetic model to illustrate the inheritance in the complex disease gene by adopting an effective statistical method that did not *a priori* assume a genetic model and avoided the problem of multiple comparisons.

The production of basal vascular wall NO is mainly determined by the key NOS 3 enzyme^40^. NO may react with reactive oxygen species to produce peroxynitrate, which can cause oxidative stress associated with neurodegenerative diseases including Alzheimer’s disease^41^. Therefore, NOS 3 enzyme involved in oxidative stress plays an important role in the pathophysiology of AD development. Evidence has been obtained showing that the G894T polymorphism in the *NOS3* influences NOS3 activity and basal NO production^42^,^43^. Increased expression of NOS3 results in altered mitochondrial function in neurons^44^. It may be explained that the polymorphism is involved in AD development through modifying the expression of NOS 3 that caused the excessive production of NO.

However, it might be argued that the identified association between *NOS3* G894T polymorphism and AD risk is due to heterogeneity. Therefore, subgroup analyses were performed to explore the stability of the association.

It is well established that *APOE є4* plays an important role in the pathogenic mechanism of AD by regulating the formation of Aβ and the *APOE є4* is the only established genetic risk factor for AD^45^. Only 10 studies performed the stratification analysis to evaluate the association between *NOS3* G894T polymorphism and AD risk by the *APOE є4* status in the included studies. When stratified by *APOE є4* status, the results from stratification analyses showed that the significant differences in genotype (overdominant model) of the polymorphism in the *APOE є4(+)* sample and the *APOE є4(−)* sample were disappeared between case group and the control group. This negative result may be because that few studies stratified by *APOE є4* status were included in the meta-analysis or *APOE є4* actually exerted an effect on association between *NOS3* and AD. Subsequently, we performed a comparison on risk of *NOS3* G894T polymorphism for AD development between *APOE є4(+)* group and *APOE є4(−)* group to explore the potential effect of *APOE є4* status. However, the statistical difference for risk comparison between the two subgroup (*P*=0.925) was not found, which excluded the possibility that *APOE є4* status exerted an effect on association between *NOS3* and AD, and speculated that the negative result may be yielded by lowed power of few included studies.

Age is very important factors for AD development. Most often, AD is diagnosed in people over 65 years of age, and every five years after the age of 65, the risk of the disease incurrence approximately doubles^46^. Moreover, some authors also reported that AD mainly affected 10% of the population with a predilection for more than 65 years^6^. Results from the stratification analysis showed that the AD risk was associated with late onset with a significant overdominant model (OR = 1.33, 95%CI = 1.07, 1.66, *P *= 0.01). Further, the findings from heterogeneity analysis showed that there was a statistical difference among age groups (P = 0.049). Therefore, the *NOS3* G894T polymorphism may be age-dependently associated with AD risk. In fact, a possible explanation for the age-dependent association could be the different plasma homocysteine level, an important factor in AD pathogenesis, as the onset time of AD was affected by the heterologous amino acid^47^. The findings from Italian study demonstrated that plasma homocysteine levels were significantly increased in late-onset AD compared with early-onset AD^29^. Moreover, plasma homocysteine levels were closely associated with *NOS3* genotype^29^. Therefore, the mechanism by which the different genotypes may affect plasma homocysteine levels may explain the age-dependent association.

The different ethnic background is a well-known confounding factor in genetic studies. Variations in the frequency of *NOS3* T allele among different ethnic groups have been reported. Evidence has been shown that *NOS3* T allele was more common in Whites than in African Americans or Asians^48^. The results from our study showed that distribution frequency of the *NOS3* T allele was 42.71–70.81% in Caucasian populations, which was lower than that in Asian populations (69.24–92.56%). However, the statistical difference was not found in Caucasian populations with an overdominant model (OR = 1.15, 95%CI = 0.99–1.34, *P ***= **0.075) and in Asian populations with an overdominant model (OR = 1.31, 95% = 1.03–1.67, *P *= 0.03) for AD risk in the stratified analysis by ethnicity (*P *= 0.246).

There are some limitations: **(i)** we had no ability to ascertain whether studies included in our review had an adequate sample size. The Genetic Power Calculation often is a measure to evaluate the sample size in genetic analysis on association between polymorphisms and diseases while no study reported an a priori sample size calculation in the included studies. Inadequate choice of sample size may lead to chance and exaggerate (or dilute) the association between *NOS3* and AD. **(ii)** It is a pity that the stratification by gender could not be performed in the meta-analysis because of unavailable information in the included case control studies. It is well known that the gender is as an important factor as the age, and gender-dependent genetic effects on risk of AD have been reported in many studies. Kim *et al.* reported that a significant increased risk of AD was found in female subjects (OR = 4.4, 95%CI = 2.4–8.0) with the *APOE є4* variant genotypes while not in male subjects (OR = 0.6, 95%CI = 0.1–2.2)^49^. In addition, Zou *et al.* showed the rs688T/T genotype of *LDLR* gene was associated with increased AD odds in males (recessive model, OR = 1.49, 95%CI = 1.13–1.97, uncorrected p = 0.005), but not in females^50^. Therefore, it needs to explore the effect of gender on association between *NOS3* and AD in the future studies.

There are some strengths: **(i)** the association on the *NOS3* polymorphism and AD has been reported in many studies; however, because of limitation of sample size, the power may be lower to assess the *NOS3* G894T polymorphism and AD risk. Therefore, the meta-analysis is a good measure to explicitly explore the effect of *NOS3* G894T polymorphism on the AD incurrence. **(ii)** we assessed studies comparing the genotype and allele distribution of *NOS3* G894T polymorphism in case group with these in various control types separately because individual control types may address different questions. Hospital-based population as a control is intended to be compared with the study group as a convenience sample. However, the limitation of the hospital-based population is inability to generalize the results to other populations and yield the selection bias. In our meta-analysis, population-based control type was used in 19 of 23 studies (83%) and a significantly statistical difference was found in the subgroup analysis (P = 0.018), which suggested the ability to generalize the results to other populations. Thus, the result has a robust stability to support the association. **(iii)** compared to other meta-analyses, more studies with larger sample size were included to make the result powerful and an effective method for meta-analysis of molecular association studies was adopted to pool data in a way that reflects the biology of gene effects and genetic models in our study.

In conclusion, result from our meta-analysis might provide evidence that the G894T polymorphism of *NOS3* is associated with AD susceptibility, especially late onset AD susceptibility. The findings from the meta-analysis on the genetic polymorphism of *NO*S3 G894T may be used to potentially evaluate individual susceptibility and explore the effective measures of control and prevention for AD.

## Additional Information

**How to cite this article**: Liu, S. *et al.* The nitric oxide synthase 3 G894T polymorphism associated with Alzheimer's disease risk: a meta-analysis. *Sci. Rep.* **5**, 13598; doi: 10.1038/srep13598 (2015).

## Figures and Tables

**Table 1:** Study characteristics from included studies in the meta-analysis.

First author	Year	Country	Ethnicity	Cases				Controls			Criteria for AD diagnosis	Time of AD onset	Control type	Quality score
				N^a^	Age^b^	Age^c^	Gender^d^	N	Age^b^	Gender^d^				
Dahiyat M -1	1999	UK	Caucasian	260	57.0	79.8	35.6	207	71.0	47.1	NINCDS/ADRDA	Mixed	PB	10
Dahiyat M -2	1999	UK	Caucasian	179	74.0	82.0	51.4	186	77.0	50.0	NINCDS/ADRDA	Late	PB	10
Crawford F	2000	USA	Caucasian	287	73.0	—	33.0	120	73.3	42.0	NINCDS/ADRDA	Late	PB	8
Higuchi S	2000	Japan	Asian	411	—	76.0	27.3	350	75.7	27.7	NINCDS/ADRDA	Mixed	PB	10
Kunugi H	2000	Japan	Asian	172	—	74.1	34.3	165	57.0	41.2	NINCDS/ADRDA	Mixed	HB	6
Emahazion T	2001	Scotland	Caucasian	121	—	—	—	152	—	—	NINCDS/ADRDA	Early	PB	11
Sanchez-Guerra M	2001	Spain	Caucasian	301	71.5	75.2	34.0	309	80.3	29.0	NINCDS/ADRDA	Mixed	HB	7
Singleton A	2001	UK	Caucasian	212	79.8	—	33.3	106	78.2	47.6	NINCDS/ADRDA	Late	PB	9
Tedde A	2002	Italy	Caucasian	220	65.0	—	36.4	95	80.8	42.1	DSM-IV	Mixed	PB	9
Kalman J	2003	Hungary	Caucasian	51	—	76.0	31.4	51	72.0	47.1	NINCDS/ADRDA DSM-III	Late	PB	7
Monastero R	2003	Italy	Caucasian	149	69.7	72.0	38.5	149	73.3	38.5	NINCDS/ADRDA	Mixed	PB	10
Yang Z	2004	China	Asian	68	72.8	—	38.2	158	71.2	50.0	NINCDS/ADRDA DSM-III	Late	PB	11
Blomqvist ME	2005	Sweden	Caucasian	382	76.2	—	37.1	170	73.2	46.7	NINCDS/ADRDA	Mixed	HB	7
Guidi I	2005	Italy	Caucasian	405	73.0	—	29.6	253	71.0	42.3	NINCDS/ADRDA	Mixed	HB	8
Akomolafe A -1	2006	USA	Caucasian	235	—	71.4	24.0	82	72.3	30.0	NINCDS/ADRDA	Mixed	PB	13
Akomolafe A -2	2006	Mixed^e^	Caucasian	259	—	68.3	34.0	127	71.7	59.0	NINCDS/ADRDA	Mixed	PB	10
Zhou Y	2006	China	Asian	530	—	71.3	63.0	601	71.5	55.9	NINCDS/ADRDA DSM-IV	Mixed	PB	9
Li H	2008	Canada	Caucasian	688	77.8	71.9	42.4	681	73.4	35.6	NINCDS/ADRDA	Mixed	PB	15
Styczynska M	2008	Poland	Caucasian	154	—	71.5	33.8	176	72.7	31.8	NINCDS/ADRDA	Late	PB	11
Wang B	2008	China	Asian	338	77.6	—	51.0	378	72.9	58.0	NINCDS/ADRDA DSM-III	Late	PB	11
Giedraitis V	2009	Sweden	Caucasian	79	—	80.2	100	361	81.8	100	NINCDS/ADRDA DSM- IV	Late	PB	10
Azizi Z	2010	Iran	Asian	100	73.8	—	44.0	100	72.6	48.0	NINCDS/ADRDA	Late	PB	9
Ferlazzo N	2011	Italy	Caucasian	69	77.2		42.0	69	75.6	34.8	NINCDS/ADRDA	Early	PB	10

DSM: the Diagnostic and Statistical Manual of Mental Disorders; NINCDS: the National Institute of Neurological Disorders and Stoke; ADRDA: Alzheimer Diseases and Related Disorders Association; MMSE: mini-mental state examination; NA: not applicable; SNP: Single Nucleotide Polymorphism; PB = population based control; HB = hospital based control.

^a^Number.

^b^age at survey.

^c^age at onset of Alzheimer’s disease.

^d^percentage of male.

^e^participants from four countries (Canada, Germany and Greece).

**Table 2:** NOS3 G894T genotype distribution among AD cases and controls in the included studies.

First author	Cases			Controls			*P*^a^ (HWE)
	G/G	G/T	T/T	G/G	G/T	T/T	
Dahiyat M -1	136	103	21	80	112	15	**0.004**
Dahiyat M -2	95	76	8	74	91	21	0.376
Crawford F	129	134	24	61	49	10	0.971
Higuchi S	350	57	4	297	52	1	0.416
Emahazion T	48	59	14	70	68	14	0.664
Kalman J	30	15	6	22	29	0	**0.005**
Kunugi H	149	23	0	143	21	1	0.812
Monastero R	62	77	10	65	76	8	**0.017**
Sanchez-Guerra M	97	154	50	101	145	63	0.408
Singleton A	88	96	28	43	53	10	0.269
Tedde A	91	106	23	38	42	15	0.554
Akomolafe A -1	193	42	0	56	25	1	0.326
Akomolafe A -2	109	119	31	61	55	11	0.778
Blomqvist ME	199	145	38	81	77	12	0.270
Guidi I	210	154	41	110	120	23	0.228
Yang Z	56	11	1	121	36	1	0.334
Zhou Y	441	77	12	495	92	14	**<0.001**
Li H	285	316	87	292	322	67	0.108
Styczynska M	106	38	10	100	60	16	0.120
Wang B	296	40	2	299	76	3	0.441
Azizi Z	67	30	3	54	44	2	**0.039**
Ferlazzo N	28	29	12	33	25	11	0.108
Giedraitis V	37	36	6	183	148	30	0.992

HWE: Hardy-Weinberg equilibrium.

^a^*P* value for HWE test in controls.

**Table 3:** Total, stratified and sensitivity analysis of NOS3 G894T polymorphism and the AD susceptibility.

Summary	N^a^	Cases/Controls	Q Test			I^2^,%	Odds Ratio	95% Confidence Interval	*P* for Z test^c^	*P* for interaction^d^
			χ^2^	df	*P* Value^b^					
**All studies**	23	5,670/5,046	44.07	22	0.003	50.1	1.18	1.04, 1.35	**0.010**	-
Subgroup analysis										
Time of AD onset										**0.049**
Early	8	706/1,525	4.17	7	0.760	0.0	1.02	0.83, 1.26	0.834	
Late	14	2,568/2,866	37.63	13	<0.001	65.5	1.33	1.07, 1.66	**0.010**	
Ethnic descent										0.246
Caucasian^e^	17	4,051/3,294	35.04	16	0.004	54.3	1.15	0.99, 1.34	0.075	
Asian	6	1,619/1,752	7.68	5	0.175	34.9	1.31	1.03, 1.67	**0.030**	
APOE										0.925
ε4+	5	347/161	5.74	4	0.220	30.3	1.25	0.82, 1.91	0.304	
ε4-	5	560/884	9.74	4	0.045	58.9	1.22	0.95, 1.56	0.116	
HWE in controls										0.065
Yes	18	4,580/3,938	29.16	17	0.033	41.7	1.12	1.02, 1.23	**0.021**	
No	5	1,090/1,108	11.51	4	0.021	65.3	1.48	1.04, 2.12	**0.030**	
Control type										0.912
PB	19	4,410/4,149	36.99	18	0.005	51.3	1.20	1.03, 1.39	**0.018**	
HB	4	1,260/897	7.07	3	0.070	57.5	1.15	0.85, 1.54	0.367	
Date of publication										0.139
≤2003	11	2,363/1,890	21.51	10	0.018	53.5	1.09	0.90, 1.34	0.380	
>2003	12	3,307/3,156	20.37	11	0.040	46.0	1.27	1.08, 1.50	**0.005**	
Quality score										0.599
Low	10	2,660/1,970	20.04	9	0.018	55.1	1.16	0.94, 1.43	0.158	
High	13	3,010/3,076	23.75	12	0.022	49.5	1.21	1.02, 1.43	**0.031**	
Sensitivity analyses										—
Minimal	22	−/−	38.46	21	0.011	45.4	1.16	1.02, 1.31	**0.025**	
Maximal	22	−/−	39.82	21	0.008	47.3	1.21	1.06, 1.38	**0.004**	

CI: Confidence interval; PB: population-based; HB: hospital-based.

^a^Number of comparisons.

^b^*P* value of Q-test for between study heterogeneity test.

^c^*P*-value of Z-test for significant test.

^d^*P* value of Q-test for between sub-group heterogeneity test.

^e^African descents were excluded from one study by Akomolafe A *et al.*^27^.

**Table 4:** mRNA expression by the NOS3 G894T genotypes, using data from the HapMap^a^.

Population	G894T genotypes^b^	No.	Mean ± SD	P^c^
CEU	GG+TT	29	6.23±0.082	0.421
	GT	31	6.24±0.075	
YRI	GG	52	6.16±0.092	0.007
	GT	8	6.20±0.031	
Asian	GG	73	6.10±0.072	0.884
	GT	16	6.07±0.070	
ALL	GG+TT	154	6.15±0.094	0.603
	GT	55	6.18±0.100	

^a^Genotyping data and mRNA expression levels for MTHFR by genotypes were obtained from the HapMap phase II release 28 data from EBV-transformed lymphoblastoid cell lines from 209 individuals.

^b^Only 5 cases among CEU were detected with TT genotype.

^c^P value of independent t-test for between sub-group heterogeneity.
